# General resource for ionospheric transient investigations (GRITI): An open-source code developed in support of the Dinsmore et al. (2021) results

**DOI:** 10.1016/j.mex.2021.101456

**Published:** 2021-07-18

**Authors:** Ross Dinsmore, J.D. Mathews, Julio Urbina

**Affiliations:** The Pennsylvania State Un., University Park, PA USA

**Keywords:** Delta-vTEC, GPS-TEC, TEC detrending, Keogram, Python, CSD, cross spectral density, delta-vTEC, Delta of the vertical Total Electron Content, FFT, fast Fourier transform, FIR, finite impulse response, GNSS, Global Navigation Satellite System, GPS, Global Positioning System, *GRITI*, General Resource for Ionospheric Transient Investigations, HDF5, Hierarchical Data Format version 5, ISR, Incoherent Scatter Radar, MSTID, Medium-Scale Traveling Ionospheric Disturbance, SCIPS, Semi-Coherent Ionospheric Pulsing Structure, sTEC, Slant-path Total Electron Content, TEC, Total Electron Content, TID, Traveling Ionospheric Disturbance, vTEC, Vertical Total Electron Content

## Abstract

The analysis techniques and the corresponding software suite *GRITI* (General Resource for Ionospheric Transient Investigations) are described. *GRITI* was used to develop the Dinsmore et al. [2] results, which found a novel classification of traveling ionospheric disturbances (TIDs) called semi-coherent ionospheric pulsing structures (SCIPS). The any-geographic range (local-to-global), any-azimuth angle keogram algorithm used to analyze SCIPS in that work is detailed. The keogram algorithm in *GRITI* is applied to detrended vTEC (vertical Total Electron Content) data, called delta-vTEC herein, in Dinsmore et al. [2] and the follow-on paper Dinsmore et al. [3], but is also applicable to any other two-dimensional dataset that evolves through time. *GRITI's* delta-vTEC processing algorithm is also described in detail, which is used to provide the delta-vTEC data for Dinsmore et al. [3].

• We detail a keogram algorithm for analysis of delta-vTEC data in Dinsmore et al. [2] and the follow-on paper Dinsmore et al. [3].
• We detail a delta-vTEC processing algorithm that converts vTEC data to delta-vTEC through detrending that is used to provide the delta-vTEC data used in Dinsmore et al. [3].
• *GRITI* is an open-source Python 3 analysis codebase that encompasses the delta-vTEC processing and keogram algorithms. *GRITI* has additional support for other data sources and is designed for flexibility in adding new data sources and analysis methods. *GRITI* is available for download at: https://github.com/dinsmoro/GRITI.

## Graphical Abstract

**Figure:**
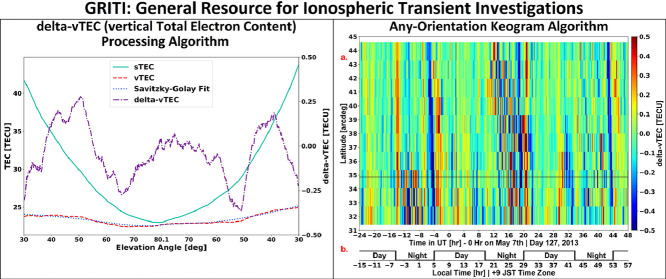

## Specifications table

**Table:** 

Subject Area:	Earth and Planetary Sciences
More specific subject area:	Ionospheric Physics
Method name:	Keogram and delta-vTEC processing
Name and reference of original method:	**For keogram:** [5] Eather, R. H., Mende, S. B., Judge, R. J. R., Plasma injection at synchronous orbit and spatial and temporal auroral morphology, J. Geophys. Res. 81 (16) (1976) 2805–2824. **For delta-vTEC processing, detrending filter method based on:** [14] Tsugawa, T., Saito, A., Otsuka, Y., Yamamoto, M., Damping of large-scale traveling ionospheric disturbances detected with GPS networks during the geomagnetic storm, J. Geophys. Res. 108 (2007) 1127, https://doi.org/10.1029/2002JA009433. [1] Coster, A.J., Goncharenko, L., Zhang, S.-R., Erickson, P.J., Rideout, W., Vierinen, J., GNSS observations of ionospheric variations during the 21 August 2017 solar eclipse, Geophys. Res. Lett. 44 (12) (2017) 041–048, https://doi.org/10.1002/2017GL075774.
Resource availability:	https://github.com/dinsmoro/GRITI

## Introduction

We give a detailed outline of the essential keogram GPS/GNSS (Global Navigation Satellite System) TEC (Total Electron Content) analysis path we implemented on the way to the results given in Dinsmore et al. [2]. Additionally, also described in detail is the *GRITI* (General Resource for Ionospheric Transient Investigations) delta-vTEC (vertical TEC) processing algorithm, which is used in an upcoming paper [3]. The resultant *GRITI* open-source code is an end-to-end analysis vehicle for the GPS/GNSS TEC datasets found in the Madrigal database (http://cedar.openmadrigal.org/). The software framework we describe supports the retrieval of absolute (total TEC magnitude in TEC units) GPS TEC data from the Madrigal database and the processing of those data into the detrended delta-vTEC (differential-vertical projection) product without any user intervention. We explain the filtering algorithms used in this processing path as well as the data format of the delta-vTEC product. The code also includes flexibility to add, analyze, and compare data from other instruments and instrument clusters. Currently, the Haystack incoherent scatter radar (ISR), the NASA OMNI data, the Kp-index data, AMPERE-derived products, and the Canadian magnetometer network are supported in our code. The OMNI and Kp-index datasets support user-intervention-free dynamic retrieval, as well. *GRITI* is a vehicle for the analysis of a single data type or for the comparison of disparate data sources and is designed to be flexible and expandible.

The GPS TEC, delta-vTEC, and other instrument data can be fed into various analysis algorithms we provide that can be used to investigate individual or ensemble results. A key piece of the analysis/display paradigm utilized in Dinsmore et al. [2] and Dinsmore et al. [3] is the keogram [5]. Note that keogram in this context means a generalized form of the classic keogram, and the generalized keogram is defined more thoroughly in the *Analysis: Keogram algorithm* section. Other analysis methods such as averaging ensemble (multi-receiver) delta-vTEC within a specified radius around a geographic point (e.g., Millstone Hill) to a single “pixel” time series, movie making, snapshot making, fast Fourier transform (FFT), and cross spectral density (CSD) techniques are supported by the code as well. To summarize, our open-source codebase allows for one-click analysis of ionospheric events with GPS/GNSS TEC and other instruments. We will support users of *GRITI* as best as possible and hope that all users will document and share enhancements on the online repository.

## Data preparation: Delta-vTEC processing

*GRITI* supports the Madrigal-format GPS TEC database which supplies absolute GPS vTEC data (plus universal time (UT) time, pierce-point location, and many other parameters) for each satellite pass over each contributing receiver over the whole Earth for a UT day. A single data day may include 80-100M data points. These data may be accessed from cedar.openmadrigal.org using *GRITI*. For Madrigal's logging and security purposes, the user needs to provide a name, email, and affiliation. Alternatively, the Madrigal website allows click-through to download data as well as various code packages to build your own process. *GRITI*, without any user intervention, processes the absolute GPS vTEC along each pass at each receiver into the delta-vTEC product which is stored in an HDF5 (Hierarchical Data Format version 5) container with other necessary parameters.

As pass-level absolute TEC data is downloaded from Madrigal on a UT whole-day basis, those passes that extend before or beyond the current UT day(s) boundary are incomplete. *GRITI* automatically completes these passes by also downloading the data from the edge days before and after the date(s) of immediate interest to complete the data for otherwise incomplete passes. Use of complete passes helps to prevent edge filtering issues when processing absolute vTEC to delta-vTEC. A permissive flag allows for incomplete pass data to be processed, even if it is missing one or both of its edge days.

The Madrigal data comes in an HDF5 format file that, due to its data structure, suffers from very slow read performance when using the Python implementation of HDF5, h5py. To sidestep this performance issue, a one-time conversion to a more efficient HDF5 structure is performed. This sidestep may not be needed when using Python with future h5py updates or for other code languages with different HDF5 implementations.

Important timekeeping stewarding/repairs also occur during the HDF5 reformatting. The need for this arises due to different timekeeping formats in different datasets and to extending GPS passes across day boundaries. For example, timestamps on day 128, h 24, min 60, and s 0 are actually (by our timekeeping standard) day 129, h 0, min 0, s 0 and should not be included in the day 128 dataset. The best approach to consistent timekeeping is to enforce the removal of all h 24, min 60, and s 60 values. Due to the cyclical nature of timekeeping, a sorting process that steps through the seconds, then the minutes, then the hours, and then the days is sufficient to move all of the timestamps onto a consistent system that never has two values representing the same time (like min 1, s 0 and min 0, s 60). The resultant unified timestamps that fall outside the analysis day are then easily identified and attached (or removed) to the correct day.

The data for the day(s) being processed as well as data from the previous and following day need to be loaded into memory. The entirety of the edge day data is not needed, though. A cutoff of six hours for the edge days will include any possible satellite observations that continue across the day boundary while reducing the memory usage.

**Fig. 1:**
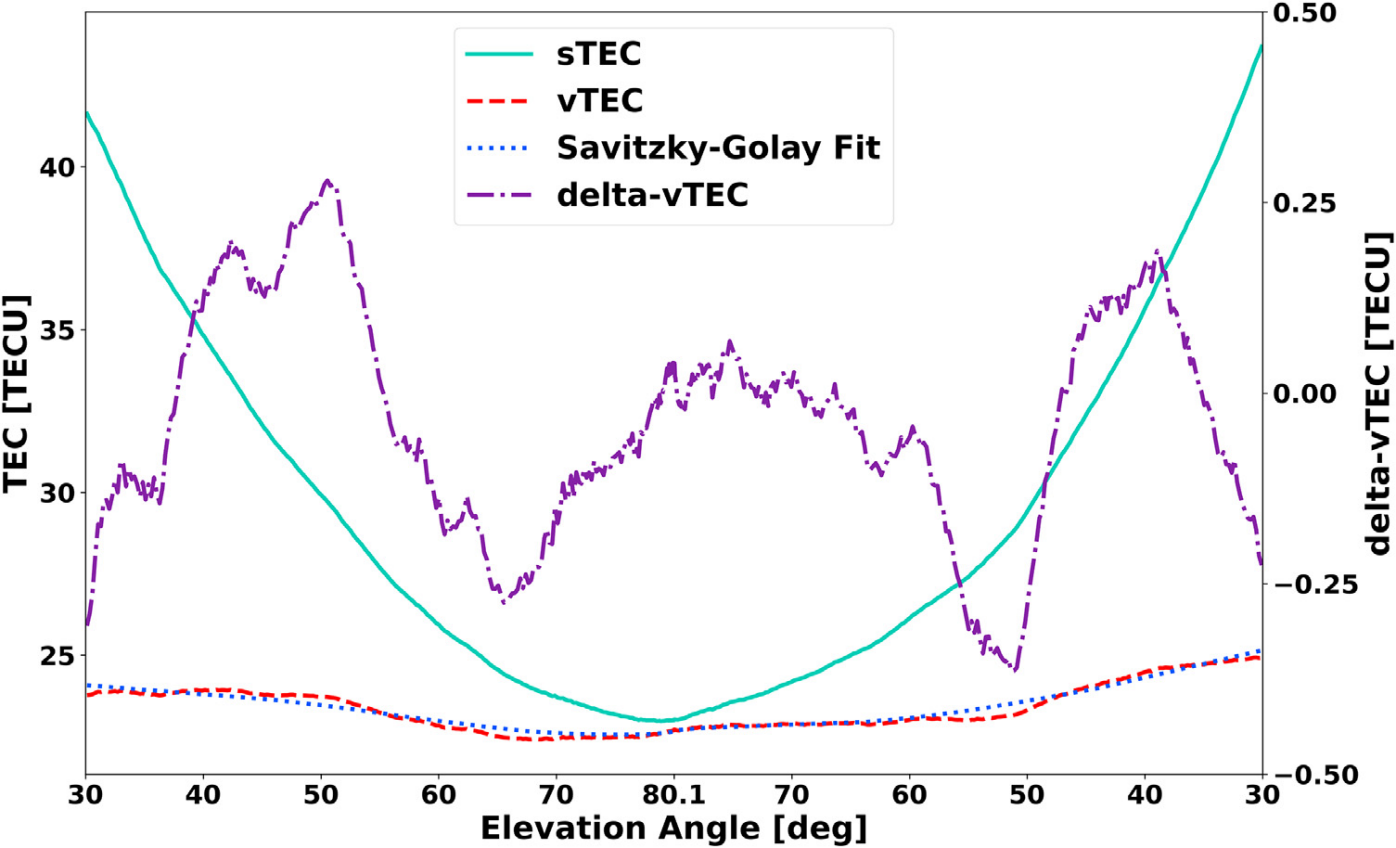
The delta-vTEC product is based on the difference between the vTEC and Savitzky-Golay fit. The TEC data products sTEC, vTEC, and Savitzky-Golay Fit use the left axis while the delta-vTEC product uses the right axis. The sTEC product is not directly used by the algorithm as the Madrigal database provides the elevation-normalized (mapped) vTEC product; it is provided for a more complete picture of the data processing path. The vTEC product is based on the sTEC product but has the bias from elevation angle removed through a process called mapping. The mapping process used by the Madrigal database is based on the method described in Rideout and Coster [10]. The vTEC data is smoothed, as described in the text, by applying a Savitzky-Golay filter [11] to the vTEC data product. The appropriately smoothed vTEC result is subtracted from the original, producing the delta-vTEC product that is then further employed in *GRITI*. Note that each delta-vTEC data point is identified with its latitude, longitude, and time parameters. The example data results shown here are for satellite G16 over receiver 0mly for the time range 2013, May 5th from 11:38:00 to 15:32:00 at approximately 58 arcdegrees latitude and 12 arcdegrees longitude near Göteborg, Sweden.

The data is farther pruned by removing any data below a user-specified minimum elevation angle value. GNSS signals pass through more of the ionosphere at lower elevation angles due to the curvature of the Earth, which leads to larger errors in the vTEC value [8]. The ionosphere effect in the slant TEC (sTEC), which is seen in Fig. 1, is already removed to produce vTEC through a process called “mapping,” which is described in Rideout and Coster [10]. The sTEC, vTEC, and delta-vTEC values are all assumed to originate at the effective ionosphere altitude (often called the pierce point altitude) used in the mapping process, which is 350 km for the Madrigal TEC database. The geographic point where the GNSS satellite to receiver path is at 350 km in altitude is used as the geographic point for each TEC measurement. The code defaults to a strict 30° minimum elevation angle, though lower elevation angles can be specified by the user.

With the desired day and edge-day data in place and the timestamps of all data aligned, sorting and filtering of the geographically appropriate GPS vTEC data along individual passes at each receiver can begin. Filtering proceeds on a receiver-by-receiver basis for a given day. For each receiver, all satellite passes are examined to verify if the pass is long enough for the selected filtering time. If so, the detrending filter is applied to that single satellite pass with typical results given in Fig. 1. The detrending method used in this code is an application of a Savitzky–Golay filter [11]. This is a low-pass FIR (finite impulse response) filter that effectively smooths the data, allowing for the difference to be taken between the original data and the smoothed (filtered) data yielding the delta-vTEC product. The Savitzky-Golay filter has been used previously in other works for TEC detrending [1,16].

The key user-controlled parameters of the Savitzky-Golay filter are the polynomial order employed and the window length. The polynomial order used here and in other works [1,16] is 1, which is a form of a running linear fit. The window length is based on the chosen filter cutoff period divided by the TEC data rate, as shown in Eq. (1):

(1) $$SG\;window\;length=\left[round\left(\frac{cutoff\;period}{TEC\;data\;rate}\right)\right]$$

As the integer SGwindowlength must be odd, the Eq. (1) result must be incremented by 1 if it is even. The cutoffperiod and TECdatarate must be the same time units. The Madrigal GNSS data interval is typically 30 s but some passes are given on 15 s or 60 s intervals in which case those passes are interpolated to (our) standard 30 s intervals. We typically employ a 121-point window corresponding to 1 h of 30 s sampled vTEC data.

**Fig. 2:**
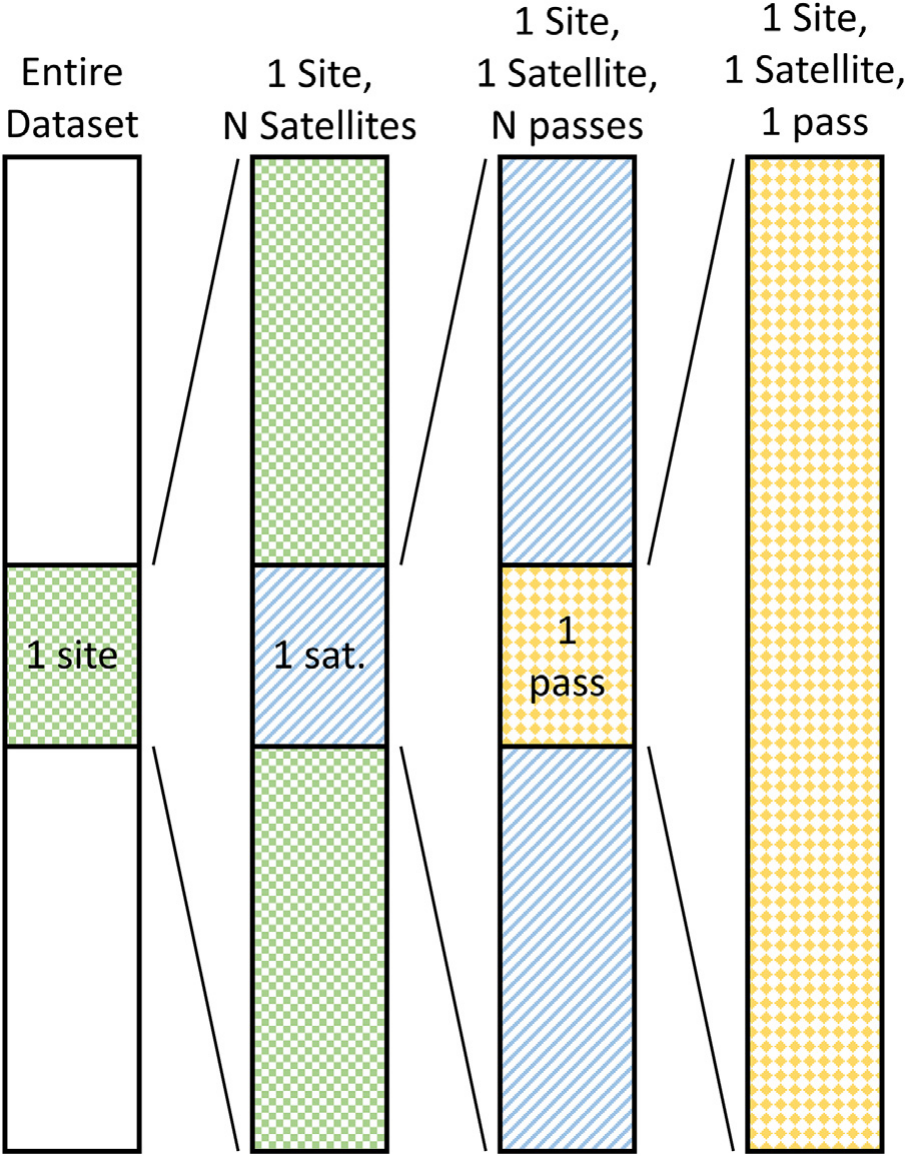
A visual representation of the steps implemented in *GRITI* to select a single satellite pass over a single receiver. Out of the large pool of many receivers (solid white, left bar), one receiver is selected (green checkered squares, left middle bar). From there, a single satellite is selected out of the pool of around 30 GPS satellites (blue slashes, right middle bar). Then a maximum time gap is used to identify the individual satellite passes over the receiver, and one of those individual passes is selected for the delta-vTEC processing algorithm to operate on (yellow checkered diamonds, right bar).

As shown graphically in Fig. 2, in order to process the data from individual satellite passes across individual receivers to delta-vTEC, the bulk 24 h UT data is sorted by receiver and further sorted by satellite. For a specific receiver and satellite combination a time-stamp vector is formed and the individual passes are identified by a user-defined time gap limit. Finally, individual passes are appropriately cataloged for further analysis. In particular, each individual satellite pass needs to be examined for completeness—if not complete it is repaired or discarded. Finally, the filtering and delta-vTEC generation process is applied to each acceptable individual satellite pass. As noted, satellite passes will sometimes have one, or a few, missing data points in a row—as recognized by a relatively small time gap—which are filled by interpolation. The maximum gap to interpolate between is a user-defined variable, and 10 points (5 min) was used in this analysis.

Each individual satellite pass is gauged to determine if it is acceptable for filtering to the desired delta-vTEC product with the Savitzky-Golay filter. In addition to limiting gaps as mentioned above, each individual satellite pass is only processed if the total satellite pass duration is greater than twice the filter cutoff period.

To be fully acceptable for processing, individual satellite passes need to be screened for several additional validity issues:

### Duplicate timestamps

If a single satellite pass has duplicate timestamps within its respective timestamp vector (e.g. two 2 h, 2 min, 30 s timestamps for a single satellite and receiver combination), the satellite pass is discarded and its data are not used.

### Day bias alignment

If an individual satellite pass crosses a day boundary, the missing vTEC segment of that pass is located in the data for the adjacent day and attached to the pass under analysis. However, the two same-pass vTEC segments likely will have a mismatch at the day edges. This is because the model ionosphere used in the mapping of the sTEC to vTEC is different for each day as gross geophysical parameters such a Kp are different [10]. Clearly, the single satellite pass that crosses the day boundary is continuous, but the different model ionospheres used cause a relative bias that results in a distinct break in vTEC continuity. To rectify this issue, we extrapolate each vTEC segment across the boundary, determine the offset, and align both sides by applying that offset to one day to create continuity. Note that, as our goal is to determine delta-vTEC, any error in this approach is second order.

### Timestamp adjustment

If the satellite pass timestamps are within ~10% of the expected data rate but do not match, the timestamps are adjusted to the (our) standard data rate of 30 s. Timestamps occur at 0/30 s in the large majority of the data. However, near-correct (e.g., 2/32 s) timestamp values are sometimes encountered. These timestamps are simply shifted to the correct 0/30 s values which greatly simplifies later ensemble analysis procedures. This is a simple fix and does not impact the data quality. The subsequent hour and day values need to be checked for min/h/day roll-over as well.

### Timestamp interpolation

If the satellite pass timestamps differ significantly from the expected data rate, repair by interpolation is attempted. This issue occurs as a few receivers sample at a different data rate than the desired 30 s interval. Rather than discarding these, they are resampled to the 30 s (0/30 per above) interval via interpolation and/or averaging depending on if the data rate is slower (e.g., 60 s) or faster (e.g., 15 s) than the desired 30 s TEC data rate.

With each of the above checks and corrections applied, then each acceptable individual satellite pass is smoothed using the Savitzky-Golay filter. This filter requires uniform-interval data, i.e., without gaps. However, as noted above, missing delta-vTEC data points under the maximum gap length are interpolated. With the gaps suitably interpolated, the desired delta-vTEC product is found by simply subtracting the Savitzky-Golay filtered vTEC data from the vTEC data.

After all of the individual satellite passes have been filtered (smoothed) and delta-vTEC found, the “obvious” outliers are removed. The origin of these outliers is usually due to intermittent operational issues in the receiver [10]. As described in Rideout and Coster [10], the Madrigal database already has whole satellite-pass outlier rejection, but “hot pixel” single vTEC values still occur. A clamp on the maximum delta-vTEC value or a maximum multiple of the median of the distance from the median of the delta-vTEC for the individual pass are two good ways to achieve outlier rejection. On geomagnetically calm days, a clamp appears to work well, although outlier rejection does not necessarily scale with the delta-vTEC distance from the delta-vTEC median. The clamp is effective because on a calm day most major outliers are due to issues with single vTEC values.

After all of the analysis steps outlined above have been completed, the day's delta-vTEC data product along with the corresponding relevant data like time, pierce-point location, elevation angle, satellite ID, and receiver name plus other documentation is saved into a new HDF5 file for easy reuse.

## Analysis: Keogram algorithm

The per-day, HDF5-formatted, delta-vTEC product can be flexibly shuffled into several analysis algorithms available in *GRITI*. These algorithms include keogram generation with very flexible parameters, a circle-pixel (radius-around-a-point) averaging algorithm for local time-series generation, a cross-spectral analysis algorithm, an FFT algorithm, and a movie making algorithm with snapshot generation. While the other algorithms are relatively simple or rely on packages for plotting or spectral analysis, the keogram averaging algorithm is more involved. The algorithm is described in detail below.

Importantly, a keogram is canonically defined as a plot with latitude on the ordinate, time on the abscissa, and a color scale to represent auroral emission intensity, per Eather et al. [5]. A few decades later the term ewogram was introduced to represent longitude on the ordinate [4]. Herein and in the related works we follow contemporary literature (e.g. Coster et al. [1]) which generalize the term keogram to more broadly represent either latitude or longitude on the ordinate while also presenting any appropriate geophysical parameter via the color scale. The essential features of a (generalized) keogram herein are latitude or longitude on the ordinate, time on the abscissa, and any activity type for the color scale.

**Fig. 3:**
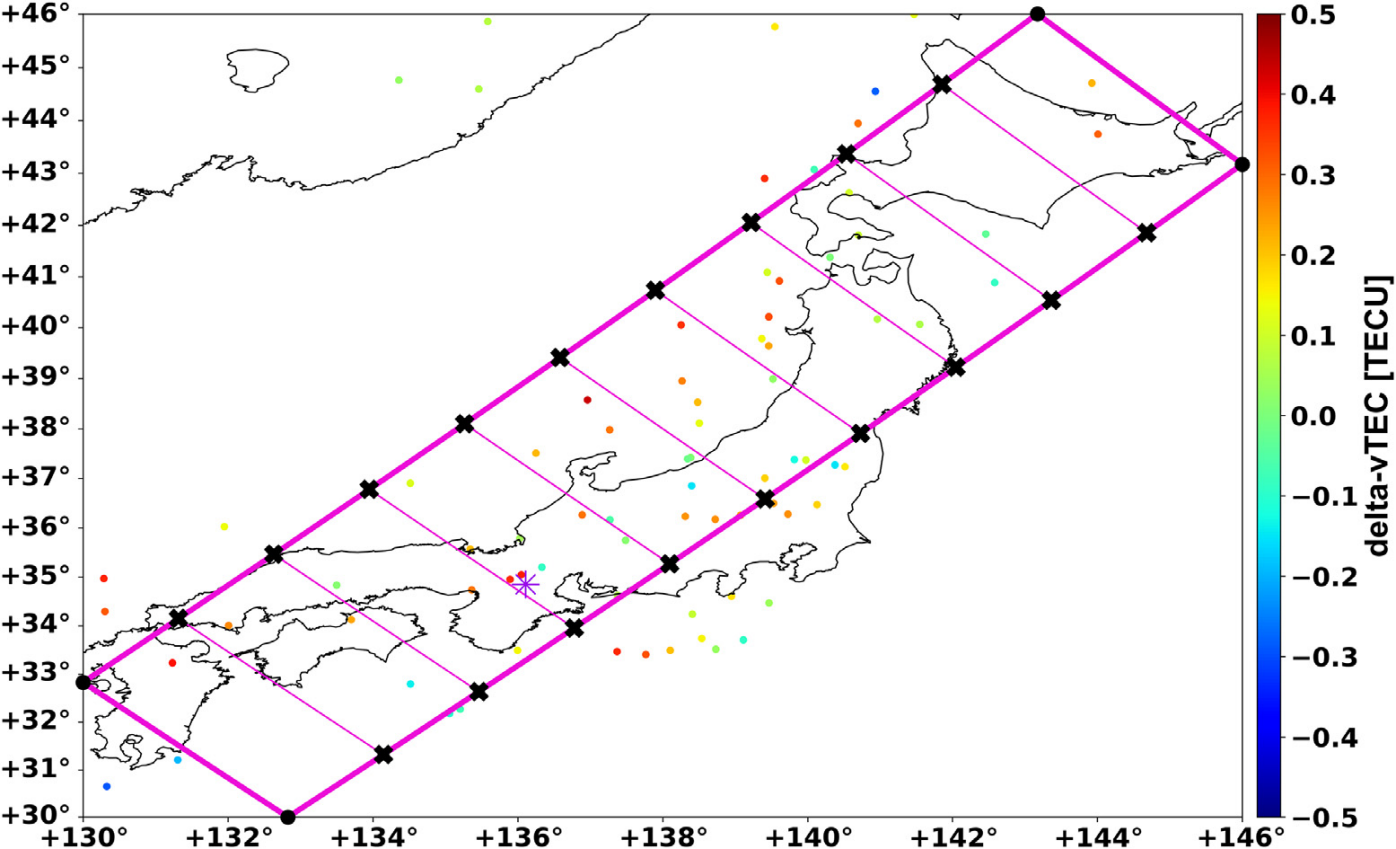
Visualization of the keogram averaging-framework. The thick magenta outer line with the round dots at the corners represents the overall keogram averaging area. For 10 or less cells, each averaging box is represented as the space between the thin magenta lines with their corresponding X points. For more than 10 averaging cells, every *n*th line is drawn to keep the visualization from becoming too crowded. The boxes between the lines are the areas averaged for each vertical “pixel” of the keogram. The normal keogram averaging-framework visualization in *GRITI* does not include the black round dots or X's. The keogram area here is set to a 45° angle with a plot area from 30 to 46 arcdegrees latitude and 130 to 146 arcdegrees longitude around Japan. The keogram also has an averaging width of 4 arcdegrees and 10 averaging cells. The purple star represents location of the Shigaraki MU Observatory.

Keogram analysis of delta-vTEC involves several steps. First, a user-defined keogram averaging-framework is set up as seen in Fig. 3. This establishes the set of parallel rectangular boxes into which the entire full-day delta-vTEC dataset is then sorted. The sorting algorithm used is a ray tracing algorithm that is greatly accelerated via pre-calculation and pre-compilation to reduce the computational time needed as a single day of TEC data can have ~80 to ~100 million delta-vTEC points distributed around the globe. As noted in the code comments, the ray tracing algorithm is used instead of a simpler sorting algorithm for orthogonal angles (0°, 90°) because it is three times faster than the current simpler sorting algorithm implementation. In those orthogonal angle instances, the ray tracing algorithm creates an averaging area that is not based on the exact orthogonal angle, but rather an angle that is slightly perturbed from the exact angle due to the slope method used to lay out the averaging zones. A flag can be used to force the exact orthogonal angles and the slower sorting algorithm, but the differences between the two sorting methods are negligible for orthogonal angles.

A particular user-defined keogram averaging-framework is constructed based on the specified “propagation angle” (e.g., north-south, east-west, or angles in between), the total plot area (the map area to calculate the keogram within), the desired keogram averaging width, and the number of averaging cells. An example of that keogram averaging area is shown in Fig. 3 with the keogram set to a 45° angle, a plot area ranging over 30 to 46 arcdegrees latitude and 130 to 146 arcdegrees longitude (encompassing Japan), a keogram averaging width of 4 arcdegrees, and a total of 10 averaging cells. The purple star represents the location of the Shigaraki MU Observatory. The 45° keogram angle will more effectively image phenomena propagating at a 45°/255° angle than orthogonal keogram angles; see Dinsmore et al. [2] Figs. 5 and 6b. for simulated medium-scale traveling ionospheric disturbances (MSTIDs) propagating at a 135° angle and the imaging results from a keogram angled to 135°.

The keogram averaging zone is made up of two outer parallel lines that are offset by ± half of the keogram width from a center line that goes through the center of the plot area. These two outer parallel lines define the boundary of the keogram averaging-framework. Being parallel lines, they share a slope but have different intercept values. The lines then need to be projected and then clipped to fit within the plot area. That process yields the four points that define the boundary lines, which are represented in Fig. 3 by the four black round dot corner points connected by the thicker magenta line.

With those defining four points computed, then two linearly spaced sets of points along the two outer boundary lines can be made based on the number of averaging cells chosen by the user. The two sets of points represent all of the averaging zones that the ray tracing algorithm will use to compute the individual pixels as a function of time. The keogram averaging boxes are constructed from two sets of adjacent points from each line. In Fig. 3, black X's represent the points used to define the averaging box edge corners and the thin magenta lines represent the edges. For more than 10 averaging cells, the keogram framework visualization code will start to plot every n^th^ line to keep the visualization from becoming too crowded with thin magenta lines.

**Fig. 4:**
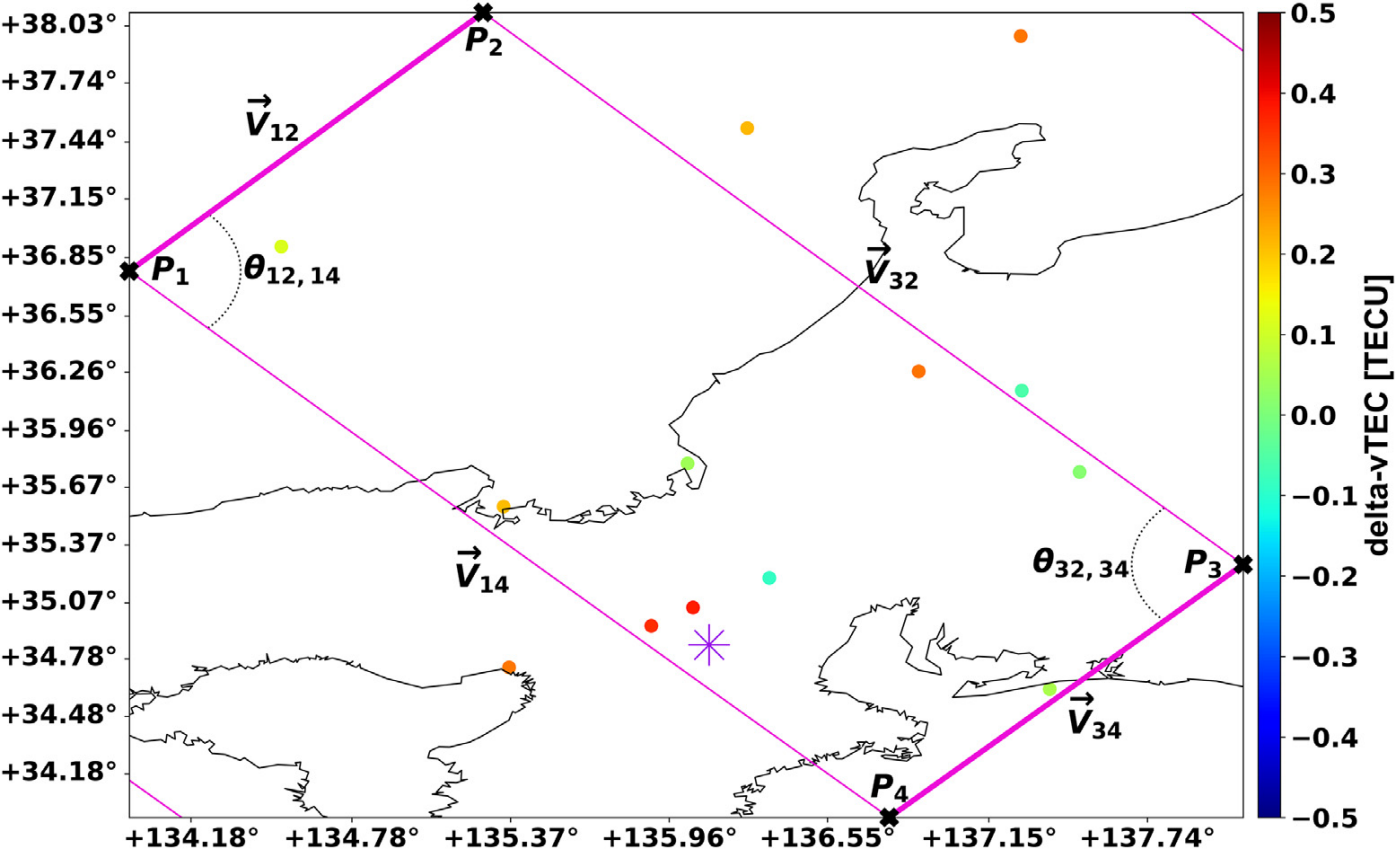
A zoomed in view of the averaging box around Shigaraki MU Observatory (represented by the purple star). Additionally, points, vectors, and angles used in the ray tracing equations are annotated. Note that the angles listed in this case are always 90° due to how the keogram averaging-framework is constructed, but the ray tracing algorithm supports parallelograms as well. The points within the box for each time interval will be identified with the ray tracing algorithm and then averaged into a single number to represent the keogram “pixel” for this box. Note the subscripting of the vectors and angles.

With the keogram averaging-framework set up, the ray tracing algorithm is applied. Ray tracing can take many forms, but since the keogram averaging boxes are rectangles, a simple five-step method is employed. This simple method assumes points 1 and 3 are opposite each other. The method employed in *GRITI* revolves around calculating the angles that make up the square and then comparing those angles to the angles that arbitrary data points have with reference to the sides of the square. Fig. 4 shows a zoomed-in view of one of the averaging boxes in Fig. 3 with the constant points, angles, and vectors noted.

Per Fig. 4:

Step 1, calculate reference vectors:

(2a) $$\overset{⇀}{V_{12}}=\left[P_{2X}-P_{1X},P_{2Y}-P_{1Y}\right]\;,\;M_{12}=∥\overset{⇀}{V_{12}}∥$$

(2b) $$\overset{⇀}{V_{14}}=\left[P_{4X}-P_{1X},P_{4Y}-P_{1Y}\right]\;,\;\;M_{14}=∥\overset{⇀}{V_{14}}∥$$

(2c) $$\overset{⇀}{V_{32}}=\left[P_{2X}-P_{3X},P_{2Y}-P_{3Y}\right]\;,\;\;M_{32}=∥\overset{⇀}{V_{32}}∥$$

(2d) $$\overset{⇀}{V_{34}}=\left[P_{4X}-P_{3X},P_{4Y}-P_{3Y}\right]\;,\;\;M_{34}=∥\overset{⇀}{V_{34}}∥$$

Step 2, calculate reference angles using the dot product:

(3a) $$\cos\left(\theta_{12,14}\right)=\frac{\overset{⇀}{V_{12}}\cdot \overset{⇀}{V_{14}}}{M_{12}M_{14}}$$

(3b) $$\theta_{12,14}=\cos^{-1}\left(\frac{\overset{⇀}{V_{12}}\cdot \overset{⇀}{V_{14}}}{M_{12}M_{14}}\right)$$

(3c) $$\cos\left(\theta_{32,34}\right)=\frac{\overset{⇀}{V_{32}}\cdot \overset{⇀}{V_{34}}}{M_{32}M_{34}}$$

(3d) $$\theta_{32,34}=\cos^{-1}\left(\frac{\overset{⇀}{V_{32}}\cdot \overset{⇀}{V_{34}}}{M_{32}M_{34}}\right)$$

These two reference angles will be used to compare to the angles calculated for each potentially enclosed data point. Steps 3, 4, and 5 are repeated for all potential data points to determine if each is inside of the keogram averaging rectangle for the time interval in question.

Step 3, calculate arbitrary point vectors where the lowercase *p* subscript refers to the relevant data coordinate:

(4a) $$\overset{⇀}{V_{1p}}=\left[P_{pX}-P_{1X},P_{pY}-P_{1Y}\right],\;M_{1p}=∥\overset{⇀}{V_{1p}}∥$$

(4b) $$\overset{⇀}{V_{3p}}=\left[P_{pX}-P_{3X},P_{pY}-P_{3Y}\right],\;M_{3p}=∥\overset{⇀}{V_{3p}}∥$$

Step 4, calculate arbitrary point angles using the dot product:

(5a) $$\cos\left(\theta_{12,1p}\right)=\frac{\overset{⇀}{V_{12}}\cdot \overset{⇀}{V_{1p}}}{M_{12}M_{1p}}$$

(5b) $$\theta_{12,1p}=\cos^{-1}\left(\frac{\overset{⇀}{V_{12}}\cdot \overset{⇀}{V_{1p}}}{M_{12}M_{1p}}\right)$$

(5c) $$\cos\left(\theta_{14,1p}\right)=\frac{\overset{⇀}{V_{14}}\cdot \overset{⇀}{V_{1p}}}{M_{14}M_{1p}}$$

(5d) $$\theta_{14,1p}=\cos^{-1}\left(\frac{\overset{⇀}{V_{14}}\cdot \overset{⇀}{V_{1p}}}{M_{14}M_{1p}}\right)$$

(5e) $$\cos\left(\theta_{32,3p}\right)=\frac{\overset{⇀}{V_{32}}\cdot \overset{⇀}{V_{3p}}}{M_{32}M_{3p}}$$

(5f) $$\theta_{32,3p}=\cos^{-1}\left(\frac{\overset{⇀}{V_{32}}\cdot \overset{⇀}{V_{3p}}}{M_{32}M_{3p}}\right)$$

(5g) $$\cos\left(\theta_{34,3p}\right)=\frac{\overset{⇀}{V_{34}}\cdot \overset{⇀}{V_{3p}}}{M_{34}M_{3p}}$$

(5h) $$\theta_{34,3p}=\cos^{-1}\left(\frac{\overset{⇀}{V_{34}}\cdot \overset{⇀}{V_{3p}}}{M_{34}M_{3p}}\right)$$

Step 5, check if the arbitrary point is inside of the rectangle:

(6) $$Is\;In?=\;\left(\theta_{12,1p}\leq \theta_{12,14}\right)\;\&\;\left(\theta_{14,1p}\leq \theta_{12,14}\right)\;\&\;\left(\theta_{32,3p}\leq \theta_{32,34}\right)\;\&\;\left(\theta_{34,3p}\leq \theta_{32,34}\right)$$

This binary check results in *true* if the point is within the rectangle and *false* if it is not. Some easy accelerations can be made based on not calculating the angle directly and just using the cosine of the angle. This requires the less-than-or-equal signs be flipped to greater-than-or-equals:

(7) $$\begin{array}{ccc} Is\;In? & = & \left(\cos\left(\theta_{12,1p}\right)\geq \cos\left(\theta_{12,14}\right)\right)\;\&\;\left(\cos\left(\theta_{14,1p}\right)\geq \cos\left(\theta_{12,14}\right)\right)\;\;\&\;(\cos\left(\theta_{32,3p}\right)\\ & \geq & \cos\left(\theta_{32,34}\right))\&\;\left(\cos\left(\theta_{34,3p}\right)\geq \cos\left(\theta_{32,34}\right)\right)\; \end{array}$$

Another easy acceleration is to apply the steps 1-5 to the outer keogram averaging rectangle that holds all of the sub-rectangles. This will allow for points that are not inside of the keogram averaging area at all to be rejected avoiding unnecessary in-the-box checks, saving computational time. Mathematical approximation can also yield some acceleration at a minor cost of location accuracy, such as using the “alpha max plus beta min” algorithm [6] to approximate the magnitude calculations by removing the computationally costly square root. These accelerations are incorporated into the algorithm, while the square root approximation is optional based on a permissive flag with the default option being non-approximated square roots.

**Fig. 5:**
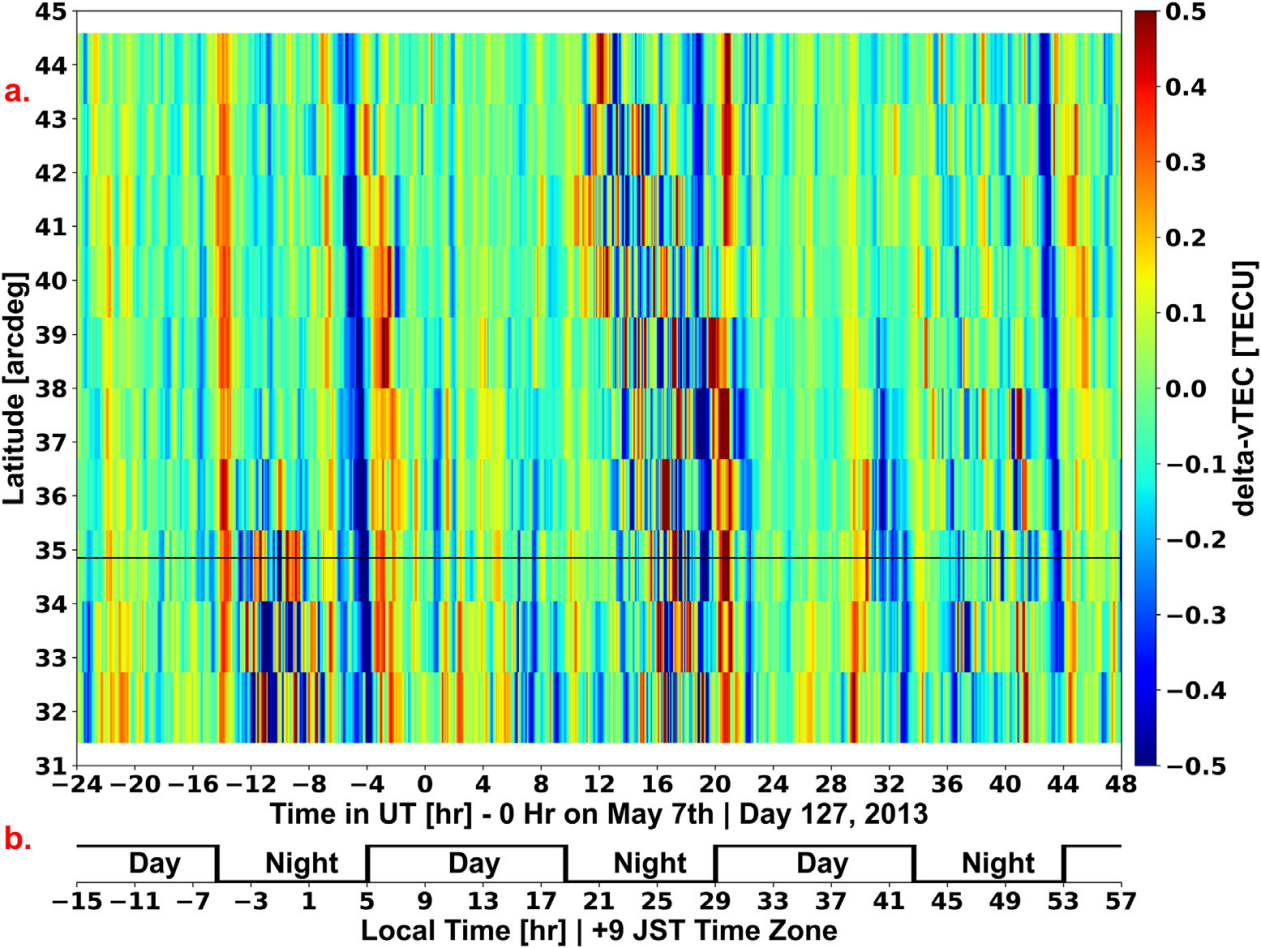
(a.) shows a keogram that is the result of the keogram averaging-framework visualized in Fig. 3. The night-to-day transitions are immediately identifiable as near-vertical blue lines, as well as increased nighttime activity. (b.) shows the local day and night periods for the Shigaraki MU Observatory at local time.

The resulting array from running the ray tracing sorting algorithm with the keogram averaging-framework on all of the delta-vTEC data within the specified time range is the keogram that can then be plotted or analyzed as needed per the results in Dinsmore et al. [2]. Fig. 5 shows the resulting array from the specifications visualized in Fig. 3, plotted as a keogram with a reference black line showing the latitude of Shigaraki MU Observatory and with an additional local time overlay localized to the Shigaraki MU Observatory's time zone. Note that for a 45° (and 135°, 225°, 315°) keogram angle *GRITI* has a user-defined flag that plots either latitude or longitude on the keogram's vertical axis, since for either case the plotted keogram looks the same. Additionally, there is no functional difference between either 45° or 225° and 135° or 315°, since the keogram averaging-framework is identical.

## Summary

The detailed algorithm for keograms, which is used in Dinsmore et al. [2] and Dinsmore et al. [3], and for delta-vTEC processing, which is used in Dinsmore et al. [3], are just a subset of the open-source *GRITI* analysis pipeline. *GRITI* also supports other data sources, like NASA's OMNI data, as well as other analysis methods like CSD or simpler averaging around a user-defined radius at a geographic location. *GRITI* is available for download and is validated to run using Python 3.

## Declaration of Competing Interest

The authors declare that they have no known competing financial interests or personal relationships that could have appeared to influence the work reported in this paper.
